# The effect of warning signs on the presence of snare traps in a Ugandan rainforest

**DOI:** 10.1111/btp.13088

**Published:** 2022-03-19

**Authors:** Pawel Fedurek, John W. Akankwasa, Dariusz P. Danel, Samuel Fensome, Klaus Zuberbühler, Geoffrey Muhanguzi, Catherine Crockford, Caroline Asiimwe

**Affiliations:** ^1^ Division of Psychology Faculty of Natural Sciences University of Stirling Stirling UK; ^2^ Budongo Conservation Field Station Masindi Uganda; ^3^ Department of Anthropology Ludwik Hirszfeld Institute of Immunology and Experimental Therapy Polish Academy of Sciences Wroclaw Poland; ^4^ School of Psychology and Neuroscience University of St Andrews St Andrews UK; ^5^ Department of Comparative Cognition University of Neuchâtel Neuchâtel Switzerland; ^6^ Department of Human Behavior, Ecology & Culture Max Planck Institute for Evolutionary Anthropology Leipzig Germany; ^7^ Institut des Sciences Cognitives CNRS Lyon France

**Keywords:** conservation, *Pan troglodytes*, poaching, snare setting, warning signs

## Abstract

Since chimpanzee (*Pan troglodytes*) conservation often involves local human populations, conservation strategies must consider psychological factors that impact their behavior. In Budongo Forest, Uganda, for example, local communities commonly engage in snare trap (hereafter: snare) setting for wild meat. This illegal activity posits a substantial threat to wild chimpanzees, causing permanent wounds or death for those who are snared. Despite various schemes previously implemented to address snare setting—an activity that is fueled by poverty, the problem and its detrimental impact on chimpanzees persists. Here, we experimentally tested a novel intervention, a systematic display of specially designed warning signs aimed at local poachers. We monitored the presence of snares before and after introducing these signs over a total period of two years and compared it with that of a similar sized control area with no intervention. Results show that snares were less likely to be present during the “sign” period than during the “non‐sign” period in the experimental but not in the control area. We discuss the potential of this cost‐effective intervention for limiting illegal activities that pose a severe threat to chimpanzees and other species inhabiting tropical forests.

## Graphical abstract

Setting snares posits a substantial threat to wild chimpanzees. We tested a novel intervention, a systematic display of specially designed warning signs aimed at local poachers. Displaying warning signs can be a cost‐effective intervention limiting snare setting.

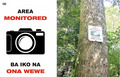

## 1 INTRODUCTION

Overexploitation of forest resources is a major threat to global tropical biodiversity (Estrada et al., 2017; Ginn et al., 2013). Indiscriminate poaching, for example, has been considered to be the “single most geographically widespread form of resource extraction” in the tropics (Fa et al., 2002). In fact, in terms of conservation challenges, biodiversity loss due to poaching is considered second only to habitat destruction and fragmentation (Vié et al., 2009). Indiscriminate poaching methods have a negative impact on population dynamics (Bunnefeld et al., 2009) and can lead to drastic population declines and extinction (Peres & Palacios, 2007). For example, many species targeted by poachers are frugivorous mammals that are essential in seed dispersal and, by extension, the health and structure of the ecosystem (Bodmer & Lozano, 2001).

Poaching activities are often fueled by poverty and population growth near the natural habitats of endangered species (Estrada et al., 2017; Ginn et al., 2013), which are often especially vulnerable to biodiversity degradation. For example, the conservation of chimpanzees (*Pan troglodytes*) is challenging in places where indiscriminate poaching and habitat destruction are practiced. Since chimpanzees are an “umbrella” species upon which many other species depend, it is important to ensure their conservation (Lambert, 2011). All four currently recognized subspecies are considered to be endangered and threatened by extinction, making them one of conservation priorities (Hockings et al., 2015; Kühl et al., 2019; Nishida, 2001).

**FIGURE 1:**
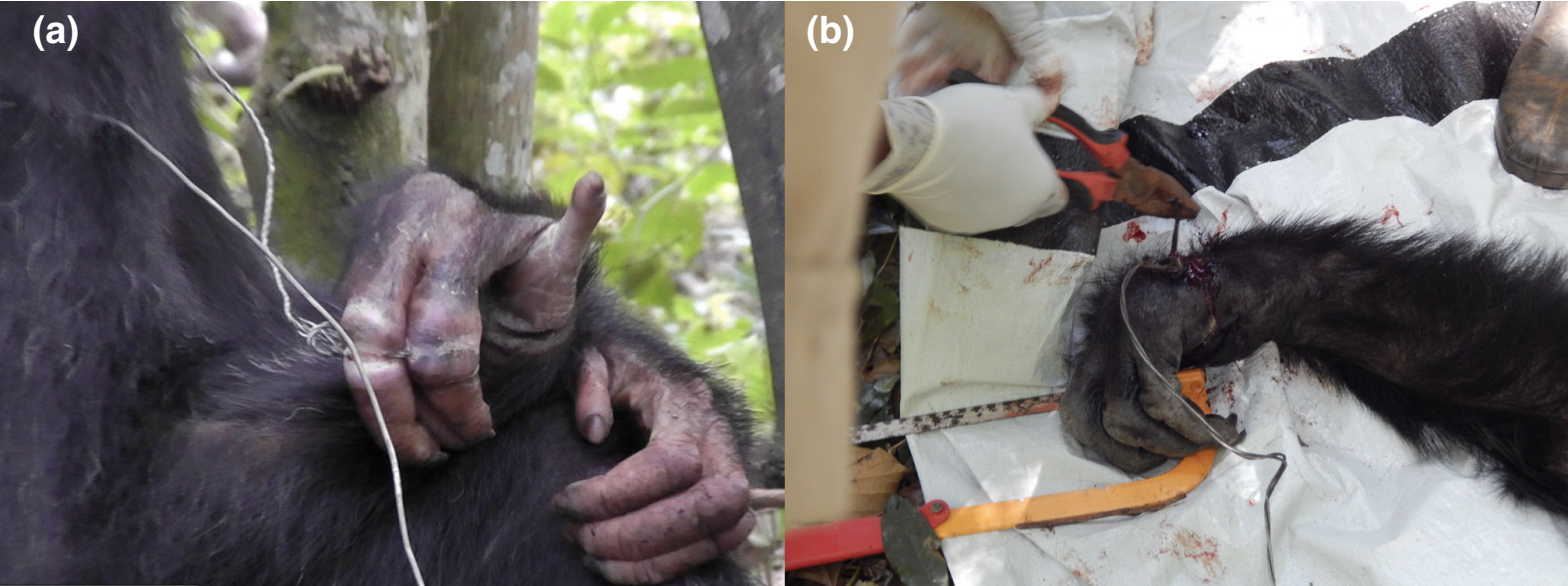
Photographs of chimpanzees injured by snares fabricated from a metal wire: (a) snare attached to a digit of an adult chimpanzee, (b) an adult chimpanzee under sedation with a snare around the wrist being removed by the Budongo Conservation Field Station (BCFS) veterinary team (Photographs by J. Akankwasa)

Wild chimpanzees are often victims of the indiscriminate poaching methods, such as the setting of “man traps” and “snare traps” (McLennan et al., 2012). In Uganda, snare traps (hereafter: snares) are easy and cheap to fabricate and commonly used by local poachers on the forest floor (Reynolds, 2006). Snares are usually made from metal wires to catch forest‐dwelling animals, such as small antelopes and pigs, for wild meat (Waller & Reynolds, 2001; Wrangham & Mugume, 2000). Although chimpanzees are sometimes hunted for meat or the pet trade (Junker et al., 2015), they are typically the unintended victims of such practices by being caught in snares, which can cause permanent injuries and death, especially in immature individuals (Munn, 2006; Quiatt et al., 2002; Reynolds, 2006; Waller & Reynolds, 2001). Chimpanzee populations inhabiting forest edges close to human settlements are particularly vulnerable. For example, in one of our study groups, the Sonso community, 26% of individuals had permanent injuries caused by snares, such as lost or crippled hand or foot, fingers or toes (BCFS, unpublished data from 2017). These injuries can considerably affect their health, wellbeing, and lifespan, a threat to wild chimpanzees across their geographical distribution (Quiatt et al., 2002) (Figure 1).

Snare setting provides a considerable challenge for chimpanzee conservation. In the past, we have implemented a number of measures with the general goal of alleviating the threats posed to chimpanzees. First, it is well established that long‐term research activities have positive conservation effects on wild animal populations (Campbell et al., 2011; Piel et al., 2015). The chimpanzees in Budongo Forest are no exception, with group sizes remaining at a consistently high level since the 1990s. The regular presence of field assistants and researchers who follow the chimpanzees reduces their exposure to poachers and their activities (Reynolds, 2005), a pattern also reported from other field sites (Wrangham & Ross, 2008). Second, we also implemented various more active intervention schemes, such as the snare removal program, also practiced by other chimpanzee research stations (e.g., Kalinzu Chimpanzee Project; Kibale Chimpanzee Project; (Asiimwe et al., 2016; Hashimoto et al., 2007; Wrangham & Mugume, 2000). At the Budongo Conservation Field Station (BCFS), the program consisted of regularly dispatching a snare removal team (SRT), comprising of former snare setters who carried out patrols throughout the home ranges of the study communities to destroy and collect snares (Asiimwe et al., 2016). In another program, we encouraged known poachers to renounce snare setting in exchange for another livelihood, that is, to breed goats as an alternative to wild meat consumption and trade. To ensure sustainability, we remained in regular contact with the participants and offered them periodically free veterinary service. BCFS has been working with about 200 renounced poachers around Budongo Forest (Asiimwe et al., 2016; BCFS, unpublished data). Finally, we regularly interacted with the local communities adjacent to the forest through outreach schemes, such as conservation‐educational workshops for both children and adults, agricultural and technical skill training, as well as events to enhance employability and health care provisioning for people and livestock (Asiimwe et al., 2016; Babweteera et al., 2018). Our educational program was designed to counteract the effects of poverty, a common driver for poaching and other illegal activities and reason for over‐reliance on small‐scale agricultural practices (Reynolds, 2005). However, despite all these efforts, snare setting and snare‐caused injuries have remained, a pattern also found in other Ugandan field stations (Wrangham & Mugume, 2000).

The purpose of this study was to implement and test a new intervention designed to reduce the number of snares being set in the forest. Specifically, we designed and displayed warning signs targeted at local poachers in a designated forest compartment for a period of one year. Our hypothesis was that the probability of encountering snares in this compartment would be higher during one year preceding the installation of the signs than in the following year during which the signs would be displayed. We adopted our approach utilizing warning signs from road traffic speed control measures that often involve displaying symbolized speeding cameras or police officers. Several studies have showed that displaying such signs by the roadside leads to drivers reducing speed (Keall et al., 2001; Lee & Sheppard, 2020). We therefore reasoned that warning signs could be also effective in other domains, such as discouraging poachers from setting snares. Warning signs have previously been effectively used in some aspects of conservation management, for example, to reduce the danger of collisions between road vehicles and wildlife (Al‐Ghamdi & AlGadhi, 2004; Found & Boyce, 2011; Khalilikhah & Heaslip, 2017; Sullivan et al., 2004; Wood & Wolfe, 1988). To our knowledge, however, warning signs have never been applied and systematically tested for their potential to influence the behavior of poachers in chimpanzee habitats.

## 2 METHODS

### 2.1 Data collection and study design

**FIGURE 2:**
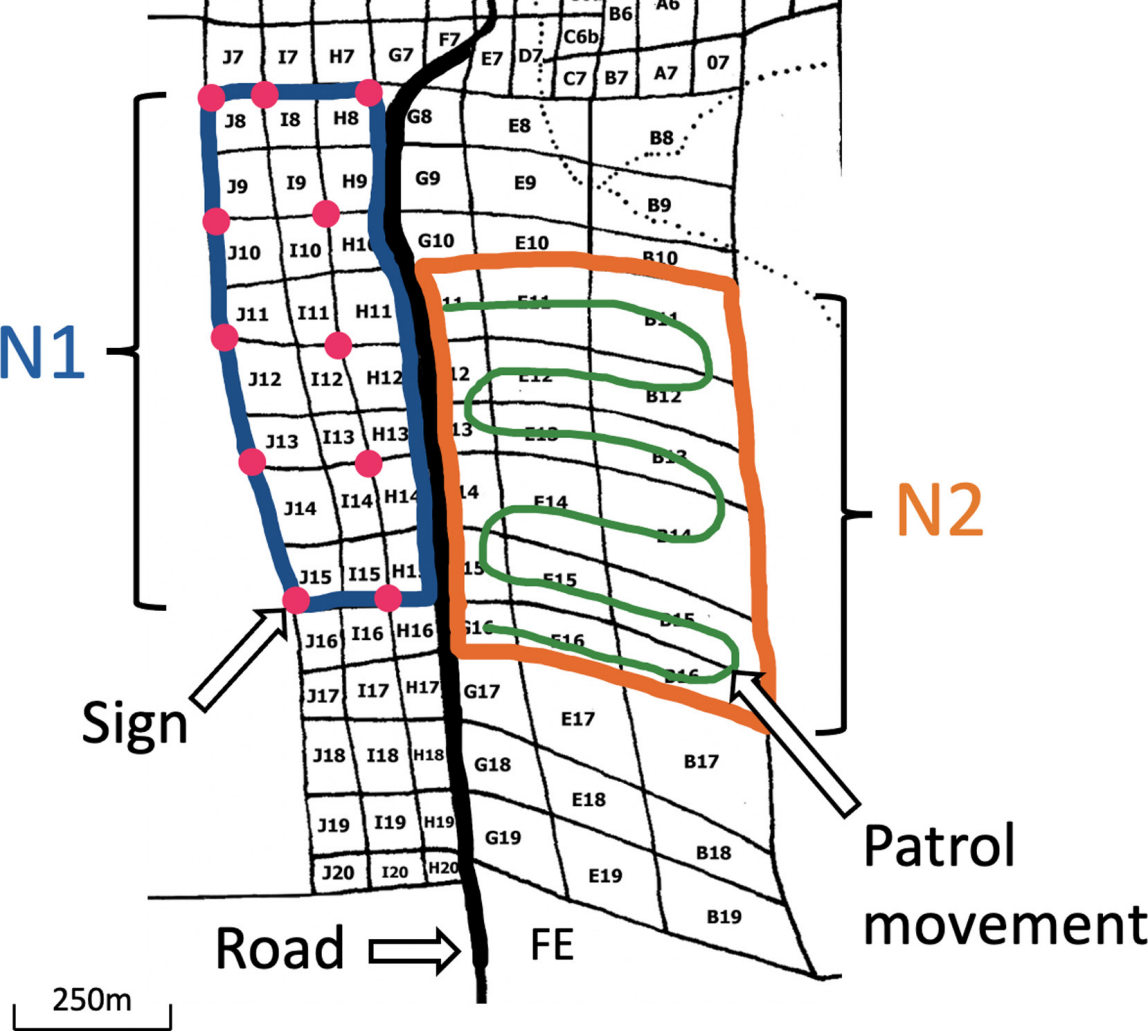
Sketch of the study area with the two compartments (N1 circled in blue, N2 in orange) showing the location of the signs (pink dots), the pattern of movement of SRT patrol in N2 (green lines), and the road separating the two compartments (thick black line). Letters with numbers indicate the names of the blocks. FE: forest edge

We collected data for two years between September 20, 2017, and October 3, 2019. We conducted the study on two compartments of the forest of comparable size, the test compartment N1 (5.4 km^2^) and the control compartment N2 (6.2 km^2^). N1 and N2 are separated from each other by the road of approximately 15 m (Figure 2). Both these compartments are within the home range of the Sonso community of chimpanzees, as well as assorted other mammal species, including forest antelopes such as blue duiker (*Cephalophus monticola*) and red duiker (*C*. *natalensis*), that are often targeted by local poachers.

**FIGURE 3:**
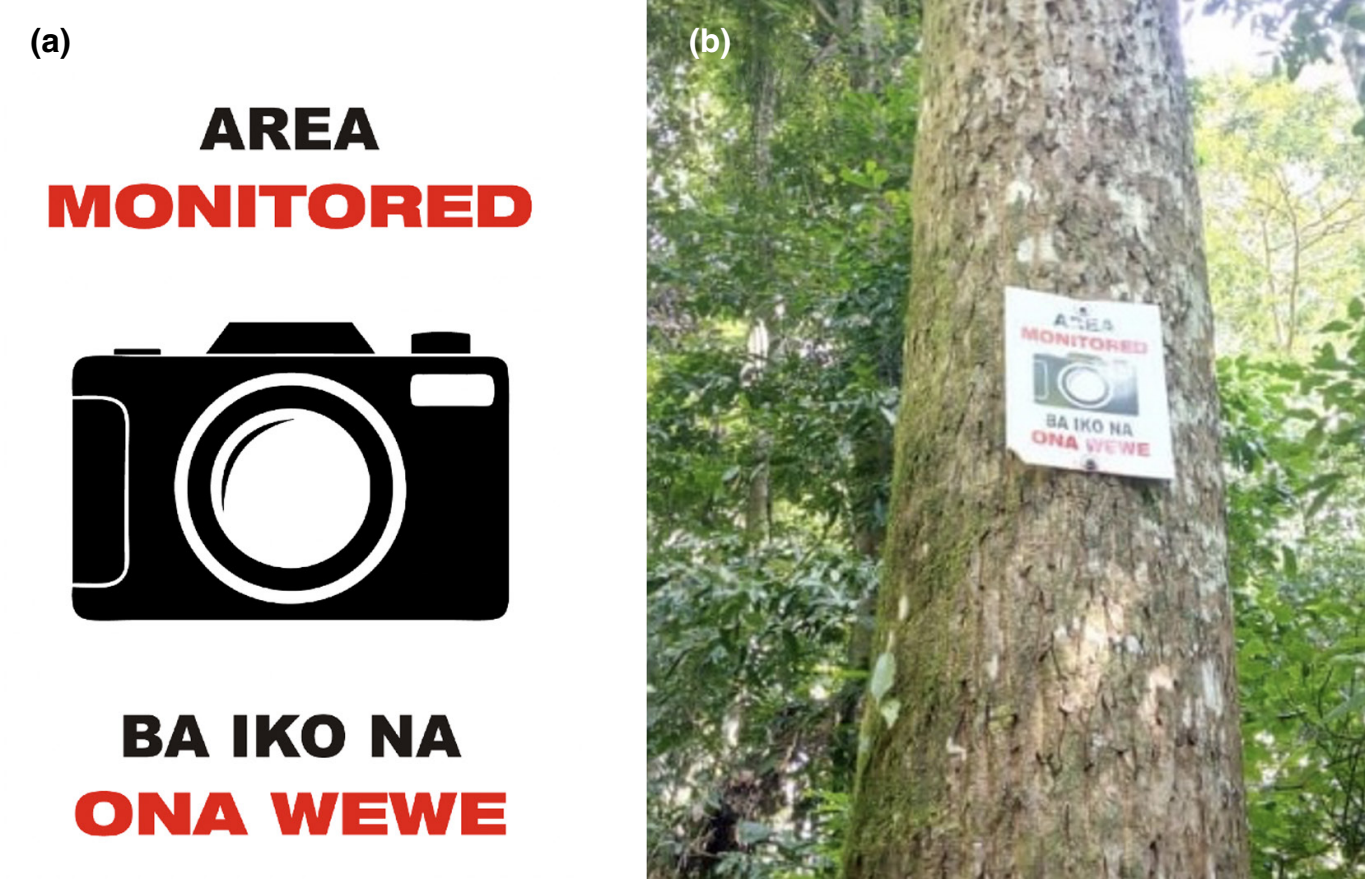
Design (a) of the warning sign used in the study and (b) one of the warning signs attached to a tree trunk

During the entire study period, every three weeks our snare removal team (SRT) comprising between 3 and 5 members conducted patrols in N1 and N2 to collect and record the number of snares found on the forest floor. The pattern of searching was the same during each patrol event. N1 and N2 comprise smaller rectangular shaped blocks separated from each other by forest trials (*N* = 24 blocks in each compartment; Figure 2). For both compartments, during each snare patrol, SRT members entered and searched each block of the compartment walking unidirectionally across the entire compartment in a zig‐zag fashion (Figure 2) with the distance between the adjacent parallel zig‐zag lines being around 100 m. This patrol strategy ensured that the compartment was thoroughly searched during each patrol. In addition, the presence and number of red duikers and blue duikers, two antelope species commonly targeted by local poachers, were recorded to examine whether the two compartments differed in terms of the number of these animals sighted there. Three‐week patrol intervals were adopted (with several exceptions where two‐ or four‐week intervals were applied to both compartments) to allow sufficient time for the accumulation of snares in the study area. In Year 2 of the study (12 October 2018 – 3 October 2019), 11 warning signs were placed in N1 but not in N2 (Figure 2). Since the number of snares in the forest can vary seasonally, with snares, for example, being more likely found during the wet season (Wrangham & Mugume, 2000; BCFS, unpublished data), non‐sign (Year 1) and sign (Year 2) periods were both one year long in duration to control for this variation. The signs were made from plastic and were 30 cm wide and 40 cm tall (Figure 3a). The signs displayed a camera in the center of the sign with a text above the camera image “Area monitored” in English and the same message below the image written in the local language (“Ba iko na ona wewe”). The signs were placed along the forest trials and attached to a tree trunk 4 m above the ground (Figure 3b).

### 2.2 Post‐experiment interviews

To establish how people from the local community interpreted the signs, after the study has been completed, we interviewed 11 adult men aged between 20 and 83 (the age‐sex group that is most likely to be involved in snare setting; Mean age = 39.82, SD = 22.08) from the adjacent to the forest village, Nyakafunjo. The interviewees were shown two photographs of the sign (Figure 3) and asked questions querying whether they understood what the image on the sign, and the sign message, meant (see Supplementary Information S1 for the questions used during the interviews).

### 2.3 Ethical note

The study was approved by the Uganda Wildlife Authority and the Uganda National Council for Science and Technology. The experiment was conducted in accordance with the guidelines of these institutions. The study was also approved by the Research Ethics Committee of Uganda.

### 2.4 Statistical analysis

In the analysis, one data point was one SRT patrol (*N* = 36 in N1, of which 18 were from Year 1 (with no signs) and 18 from Year 2 (with signs), and *N* = 36 in N2 (no signs in both years of data collection). Since there was a large proportion of datapoints with the number of snares equaling zero (likely because poachers did not enter the forest fragment during that time; Table 1) and some clear outliers (e.g., 27 snares found during a single patrol event in N2 during Year 1), generalized linear models (GLM) were used. To compare the presence of snares between Year 1 and Year 2 in the two compartments, we created two GLMs with a binomial error structure (N1 model and N2 model) (Bolker et al., 2009), one for each compartment (N1 and N2, respectively). In both models, as the dependent variable we included the presence (0/1) of snares, while as the independent variable the study period (0—Year 1; 1—Year 2). Since the probability of finding a snare might be related to the number of SRT personnel and the time spent searching, in both models we included the variable “search effort,” which represented the time (min) spent searching (N1 mean = 126.0, *SD* = 34.7; N2 mean = 140.9, *SD* = 43.6) divided by the number of people searching (which was between 3 and 5 crew members) as an additional independent variable. We used a likelihood ratio test (LRT) to test the full model against a reduced model comprising the control independent variables (searching effort).

**TABLE 1:** Presence and number (nb) of snares retrieved in N1 compartment (with signs) and N2 compartment (without signs), number of patrols, search effort, and the number of duiker sightings, during Year 1 and Year 2 of the study

Compartment	N1		N2	
Study year	Year 1	Year 2	Year 1	Year 2
Nb of snares per year	62	24	77	28
Mean ± SD nb of snares ^a^	3.44 ± 3.69	1.33 ± 2.59	4.28 ± 6.90	1.55 ± 2.64
Proportion of patrols with snares	0.78	0.39	0.61	0.44
Nb of patrols	18	18	18	18
Search effort	31.13	25.77	40.32	26.72
Nb of blue duikers	15		21	
Nb of red duikers	13		6	

^a^ Note that the mean number of snares in Year 1 in N2, and therefore the difference in mean snare numbers between Year 1 and Year 2 in this compartment, were inflated by an outlier (as shown by the large *SD* and explained in the Methods).

## 3 RESULTS

### 3.1 The probability of finding snares

**FIGURE 4:**
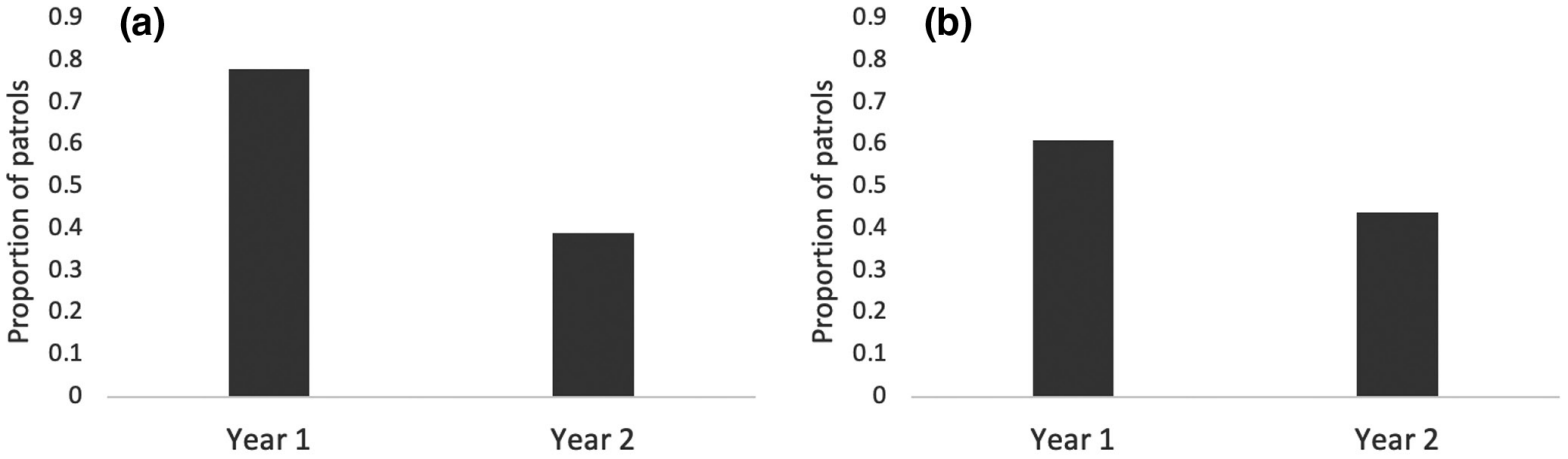
Proportion of patrols in which snares were found during Year 1 and Year 2 of the study in (a) N1 compartment with signs and (b) N2 compartment without signs

The presence and number of snares, search effort, and the number of duikers in Year 1 and Year 2 for both compartments are summarized in Table 1 (See also Figure 4).

The full model was significantly different from the reduced model when running the N1 (LRT: *p* = 0.010) but not N2 (LRT: *p* = 0.975) model.

In N1, the proportion of patrol days when snares were found was larger in Year 1 than in Year 2 (Estimate ±SD = −0.44 ± 0.17, *t* = −2.56, *p* = 0.015, 95% CI = from −0.78 to −0.10; Table 1, Figure 4a). The search efforts did not predict whether or not snares were found (Estimate ±SD = −0.06 ± 0.08, *t* = −0.73, *p* = 0.465, 95% CI = from −0.23 to 0.10; Table 1).

Although the number of red duikers and blue duikers recorded in N1 and N2 differed, the total number of duikers (two species taken together) recorded in these areas during the study period was similar (28 and 27, respectively; Table 1).

### 3.2 Post‐experiment interviews

All adult men (*N* = 11 of 11) from the adjacent to the forest Nyakafunjo village that were interviewed after the study was completed, had seen the signs, understood the camera symbol on these signs as well as that the purpose of these signs was to warn against illegal activities such as snare setting (note that in Budongo local people are allowed to enter the forest to collect firewood, fruits, mushrooms, herbs, craft materials, and other important everyday life materials).

## 4 DISCUSSION

Although a considerable number of preventive measures have been implemented over the years, snare setting remains a major conservation problem that impacts on the health and wellbeing of many tropical forest animals, including chimpanzees. Here, we tried a new method, derived from road traffic control and management: The display of symbolic signs that suggest covert automated camera‐based monitoring of the forest. We found that snares were less likely to be found by our snare‐removal experts during the period when warning signs were displayed compared to control periods and control areas. Through this comparison it is therefore possible that the warning signs had a deterrent effect on poachers, discouraging them from setting snares in the surveyed forest areas. In this respect, our findings bear similarities to results of previous studies showing that road signs displaying speed cameras induce drivers to reduce their speed (Keall et al., 2001; Lee & Sheppard, 2020), or studies showing that wildlife crossing signs reduce animal‐vehicle collisions (Bond & Jones, 2013; Found & Boyce, 2011; Khalilikhah & Heaslip, 2017; Sullivan et al., 2004).

It is clear that one of the reasons for the persistence of snare‐based poaching in Budongo is the low material costs of the activity and the low risks of being caught and prosecuted (BCFS, unpublished data). Poverty and a steady population growth in the region around Budongo Forest, fuelled by people displaced from the oil refinery and by political instability in the neighboring Democratic Republic of the Congo, are likely to be responsible for the persistence of poaching activities (Reynolds, 2005), regardless of any conservation countermeasures.

Another reason for people persistently setting snares might be due to the apparent ineffectiveness of the alternative schemes initiated by conservation agencies working in the area. For example, the alternative livelihood scheme (awarding goats to individuals that renounced poaching) implemented by BCFS led to local people questioning as to why poachers rather than non‐poachers were being rewarded (C. Asiimwe, unpublished data). This initiative, though aimed at improving the livelihoods of the vulnerable poachers, might have contributed to more people poaching to obtain rewards. Furthermore, some renounced poachers included in this initiative have been reported actively involved in poaching even after receiving the rewards (BCFS, unpublished data).

This suggests that further and more direct measures are needed to deal with the problem of poaching. We view our approach, with its deterrent effect on poachers, as an example of such a more direct intervention that should be considered when implementing new measures aimed at improving the protection of chimpanzees or other animals. An advantage of an intervention involving warning signs is that it is cost effective and complementary to other snare removal or equivalent programs already in operation. The proposed intervention, therefore, is not meant to replace other interventions aimed at reducing this problem, such as educating local communities about the harmful effect of snares on animals or schemes aimed at providing poachers with alternative to poaching means of livelihood (Asiimwe et al., 2016; Babweteera et al., 2018). Instead, our approach should be taken as an additional, although potentially powerful, intervention to the existing programs. Considering that the problem of snare setting and its harmful and often deadly effect on chimpanzees persists despite measures designed to limit it, new and more direct approaches to this old problem are urgently needed.

We are under no illusion that our measure may only have a temporary effect. We suspect that for this intervention to be successful in the long term, having at least several camera traps installed in the forest would almost certainly improve the deterrent effect of such interventions. Otherwise, we suspect that over time local communities would learn that the signs do not display true information and their deterrent effect would decrease. Indeed, studies on speed driving show that the combination of warning signs and cameras are a powerful tool in reducing traffic speeding (Pilkington & Kinra, 2005), although signs on their own can be also effective (Keall et al., 2001; Lee & Sheppard, 2020). Therefore, while our study suggests that even without cameras warning signs could be effective in discouraging local communities from setting snares, at least in the short term, future studies should evaluate the effectiveness of warning signs accompanied by camera traps (with individuals caught on camera setting snares being prosecuted) in the longer term. Camera traps could be also effective in recording more detailed data on the presence and activities of poachers in the study area, and possibly on how often poachers encounter warning signs—aspects that were not incorporated in our study. Future studies should perhaps also select the test and control compartments that are spatially separated from each other more than in our study, making data from these two kinds of study areas more independent from each other. On the contrary, it is noteworthy that, in our study, the likelihood of finding snares between the sign and non‐sign years was significantly different for the test, but not control, compartment regardless of the relatively close proximity between them. We are also aware that our sample sizes are limited, so we encourage future studies to incorporate larger or more forest compartments, or/and longer data collection periods, which would provide more detailed data on the effect of signs on both snare presence and numbers. Investigating whether signs influence the spatial distribution of snares due to, for example, poachers setting them away from the signs, is also a promising research avenue. Future studies should also systematically monitor snare presence after the signs have been removed, something that we did not investigate in our study.

Another limitation is that the patrol effort in Year 1 was greater than in Year 2. Future studies should ensure that this effort is distributed equally across the study period. However, it is noteworthy that this applied to both study compartments, suggesting that this variation in sampling effort did not have much impact on the results. Similarly, the smaller occurrence of snares recorded in Year 2 than in Year 1 could be due to the possibility of a decreased poaching activity in Year 2 compared to Year 1 in that forest area. However, the long‐term BCFS data on the number of snares recovered from the forest between 2017 and 2019 (6.9 of snares per search day in 2017, 8 in 2018, and 8.7 in 2019; BCFS, unpublished data), do not suggest a decreasing trend in poaching in the forest during the study period. On the contrary, the apparent drop in snare numbers between Year 1 and Year 2 in both compartments (Table 1) regardless of the overall rise in snare numbers in the forest during this period suggests that the signs could have affected poaching activities also in N2 due to its proximity to N1 with the signs. Again, however, the difference between the two years in terms of the proportion of patrol events during which snares were found was significant for N1 but not N2, suggesting a stronger influence of signs in N1.

Importantly, warning signs could be also effective in preventing or limiting other illegal activities in protected forests such as illegal logging. At Budongo, for example, cutting trees for charcoal production that often takes place in the forest, and that substantially damages the forest environment (Chidumayo & Gumbo, 2013), is commonly practiced by local communities (Reynolds, 2005). It is possible that such illegal activities can be deterred by warning signs in a similar way snare setting appears to be. We consider our study as a promising starting point of a new approach to the problem of snare setting and other activities harmful to wildlife that, after refining and improving it, has a considerable potential to contribute to the conservation of chimpanzees, as well as other species targeted by poachers, and their environment.

A few interventions, including those introduced by BCFS, have been implemented with the aim of conserving chimpanzees and other primate species (Asiimwe et al., 2016; Babweteera et al., 2018; Hartel et al., 2020; Reynolds, 2005). Unfortunately, however, the effectiveness of the vast majority of these interventions have not been quantitatively evaluated, or such data have not been published, and therefore, the efficacy of these approaches remains unclear (Junker et al., 2020; see also McKinnon et al., 2015). It is therefore difficult to evaluate the effectiveness of the intervention proposed in this study against previous interventions. This relates to a general problem in primate conservation, where policies or interventions are implemented without testing their effectiveness beforehand (Junker et al., 2020). For example, recent analyses suggest that such interventions have been often based on their perceived effectiveness rather than quantitative data (Junker et al., 2020). Furthermore, only around 1% of studies on primates were devoted to evaluating conservation effectiveness (Junker et al., 2020). Therefore, there is an urgent need for conservation interventions that are scientifically tested or validated prior to implementation, which ultimately would make conservation management and decisions more informed, and evidence based (Christie et al., 2020; Junker et al., 2020; Sutherland & Wordley, 2017). Regardless of its limitations, therefore, we consider our study as an example of how a new intervention aimed at conserving chimpanzees or other primates can be tested. We believe that our study will encourage further studies in this area, ultimately making interventions aimed at primate conservation scientifically more informed and rigorous.

## CONFLICT OF INTEREST

The corresponding author confirms on behalf of all authors that there have been no involvements that might raise the question of bias in the work reported or in the conclusions, implications, or opinions stated.

## AUTHOR CONTRIBUTION

PF and CA conceived the study; JWA and PF coordinated data collection; PF, DPD, and SF analyzed the data; CC, GM, and KZ provided advice and necessary resources; PF led draft writing with all authors contributing to the final version.

## Supporting information

Supplementary Material

## Data Availability

Data sets used in this study are openly available on GitHub at https://github.com/PawelFedurek/SnareProject
