# Supplementary material for: The effect of warning signs on the presence of snare traps in a Ugandan rainforest
Source: Biotropica. 2022 Mar 19;54(3):721–8. doi: 10.1111/btp.13088 (PMC9314064; doi:10.1111/btp.13088)
Supplement: Supplementary file 1 — Supplementary Material [file BTP-54-721-s001.docx]

SUPPORTING INFORMATION

**The effect of warning signs on the presence of snare traps in a Ugandan rainforest**

Pawel Fedurek ^1,2^, John W. Akankwasa ^2^, Dariusz P. Danel ^3^, Samuel Fensome^1^, Klaus Zuberbühler ^2,4,5^, Geoffrey Muhanguzi ^2^, Catherine Crockford ^6,7^, Caroline Asiimwe ^2^

^1^ Division of Psychology, Faculty of Natural Sciences, University of Stirling, Stirling, UK

^2^ Budongo Conservation Field Station, Masindi, Uganda

^3^ Department of Anthropology, Ludwik Hirszfeld Institute of Immunology and Experimental Therapy, Polish Academy of Sciences, Poland

^4^ School of Psychology and Neuroscience, University of St Andrews, St Andrews, UK

^5^ University of Neuchâtel, Department of Comparative Cognition, Neuchâtel, Switzerland

^6^ Department of Human Behavior, Ecology & Culture, Max Planck Institute for Evolutionary Anthropology, Leipzig, Germany

^7^ Institut des Sciences Cognitives, CNRS, Lyon, France

Corresponding authors:

1) Pawel Fedurek, University of Stirling, Division of Psychology, FK9 4LA, Stirling, Scotland (UK). Tel: [+44 (0)1786 467844](tel:+441786467844); email: [pawel.fedurek@stir.ac.uk](mailto:pawel.fedurek@stir.ac.uk)

2) Caroline Asiimwe, Budongo Conservation Field Station, PO Box 362 Masindi, Uganda. Tel: +256 (0)756 724 69; email: [asiimwecaroline@gmail.com](mailto:asiimwecaroline@gmail.com)

**Appendix S1:** Post-experiment questionnaire used to establish whether local people from a village adjacent to the study area understood the meaning of the signs

1. Have you ever seen this sign before?

*(Answer options: yes/no)*

*(Instruction for the interviewer: show the images of the sign. If the answer if “yes” proceed to question 2 and skip the question 5. If the answer is “no”, proceed to question 5 and skip questions 2-4)*

1. If you have seen this sign before, where did you see it?

*(Instruction for the interviewer: make a note of all the answers)*

1. And how would you understand the meaning/message of this sign?
2. Did you interpret the meaning of this sign by yourself, or someone else told you what it means?
3. If you haven’t seen the sign before, imagine that you found this sign in the Budongo forest. How would you understand the meaning/message of this sign?

*(Instruction for the interviewer: ask this question only to those who have not seen the sign before – those who answered “No” to Question 1)*

1. What does the symbol/picture on that sigh show?

*(The question refers to the camera symbol)*

1. Do you know what a camera is?

*(Answer options: yes/no)*

1. Do you think cameras can take photos of people engaging in illegal activities in the forest, such as snare setting?

*(Answer options: yes/no)*

1. Any other comments/opinion about the sign?
2. Your age:
3. Your education level:


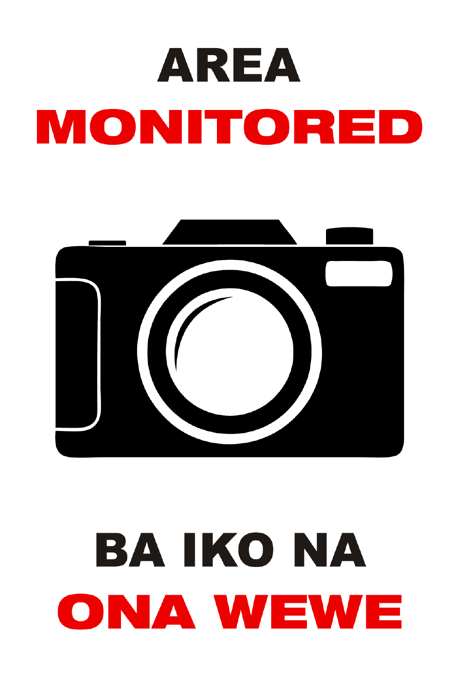


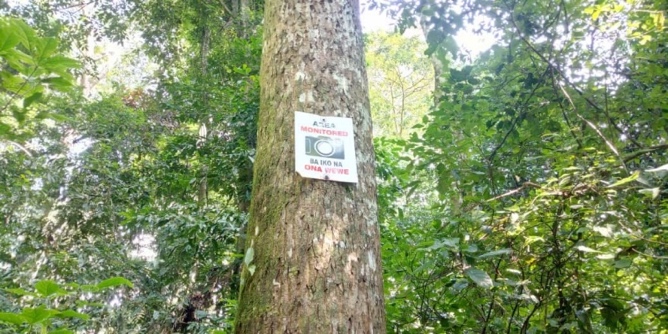


FIGURE S2 Images of the sign used in the survey
